# Neuromuscular shoulder activity during exercises with different combinations of stable and unstable weight mass

**DOI:** 10.1186/s13102-020-00168-x

**Published:** 2020-03-26

**Authors:** Omar Baritello, Mina Khajooei, Tilman Engel, Stephan Kopinski, Andrew Quarmby, Steffen Mueller, Frank Mayer

**Affiliations:** 1 Clinical Exercise Science, Department Sports and Health Science Medicine, University Outpatient Clinic Potsdam, University of Potsdam, Am Neuen Palais 10, House 12, 14469 Potsdam, Germany; 2 Computer Science and Therapy Science, Trier University of Applied Science, Schneidershof, 54293 Trier, Germany

**Keywords:** EMG, Instability, Overhead athlete, Unstable resistance training, Water pipe, Rotator cuff

## Abstract

**Background:**

Recent shoulder injury prevention programs have utilized resistance exercises combined with different forms of instability, with the goal of eliciting functional adaptations and thereby reducing the risk of injury. However, it is still unknown how an unstable weight mass (UWM) affects the muscular activity of the shoulder stabilizers. Aim of the study was to assess neuromuscular activity of dynamic shoulder stabilizers under four conditions of stable and UWM during three shoulder exercises. It was hypothesized that a combined condition of weight with UWM would elicit greater activation due to the increased stabilization demand.

**Methods:**

Sixteen participants (7 m/9 f) were included in this cross-sectional study and prepared with an EMG-setup for the: Mm. upper/lower trapezius (U.TA/L.TA), lateral deltoid (DE), latissimus dorsi (LD), serratus anterior (SA) and pectoralis major (PE). A maximal voluntary isometric contraction test (MVIC; 5 s.) was performed on an isokinetic dynamometer. Next, internal/external rotation (In/Ex), abduction/adduction (Ab/Ad) and diagonal flexion/extension (F/E) exercises (5 reps.) were performed with four custom-made-pipes representing different exercise conditions. First, the empty-pipe (P; 0.5 kg) and then, randomly ordered, water-filled-pipe (PW; 1 kg), weight-pipe (PG; 4.5 kg) and weight + water-filled-pipe (PWG; 4.5 kg), while EMG was recorded. Raw root-mean-square values (RMS) were normalized to MVIC (%MVIC). Differences between conditions for RMS%MVIC, scapular stabilizer (*SR*: U.TA/L.TA; U.TA/SA) and contraction (*CR*: concentric/eccentric) ratios were analyzed (paired t-test; *p* ≤ 0.05; Bonferroni adjusted α = 0.008).

**Results:**

PWG showed significantly greater muscle activity for all exercises and all muscles except for PE compared to P and PW. Condition PG elicited muscular activity comparable to PWG (*p* > 0.008) with significantly lower activation of L.TA and SA in the In/Ex rotation. The *SR* ratio was significantly higher in PWG compared to P and PW. No significant differences were found for the *CR* ratio in all exercises and for all muscles.

**Conclusion:**

Higher weight generated greater muscle activation whereas an UWM raised the neuromuscular activity, increasing the stabilization demands. Especially in the In/Ex rotation, an UWM increased the RMS%MVIC and *SR* ratio. This might improve training effects in shoulder prevention and rehabilitation programs.

## Background

The human shoulder is a very complex joint, characterized by a wide, multidirectional range of motion. Unlike other body articulations, its stability is mainly based on the related surrounding musculature [1–3]. Dynamic stabilization during arm/shoulder movements is obtained by synergetic mechanisms of shoulder muscles’ co-contractions, appropriate positioning, control and coordination of the shoulder/scapula-thoracic complex [4, 5]. Structures like the glenohumeral capsule/ligaments, glenoid labrum and bony geometry ensure structural stability (static stabilizers) [5]. This combination of extreme joint mobility and high neuromuscular stability-patterns, required to maintain articulation integrity, presents a significant challenge for the prevention of shoulder injuries. Accordingly, injuries are likely to occur among overhead athletes, mostly caused by repetitive above-head high speed movements [6–9]. Particularly in sport specific movements (e.g. throwing a ball), the neuromuscular control system, joint capsule and surrounding ligaments are challenged, facing high shear forces and angular acceleration placed across the joint complex [9–11].

Appropriate strengthening of the shoulder dynamic stabilizer muscles and adequate neuromuscular control-patterns is crucial in preventing shoulder injuries [4, 12]. Based on these insights, recent shoulder injury prevention programs for athletes [4, 13, 14] have proposed specific shoulder instability resistance training exercises. Particularly, most of the exercises are performed uni-laterally in unstable conditions involving an increased level of postural control (standing, planking, kneeling and laying on stability ball) and/or with external overload devices challenging motor-coordination (elastics, balls, dumbbells). Resistance training exercise promotes neural and structural modifications [15, 16] and combining them with instability can increase the sensory, biomechanical and motor-processing pattern [17]. Furthermore, neuromuscular adaptations like decreased co-contractions and improved coordination have been reported, with a consequential increase in joint stability [16, 18, 19]. Several authors have investigated the features of instability resistance training, providing evidence that stability alteration during resistance exercise effectively enhanced neuromuscular activity (e.g. mean amplitudes) despite a lower external overload involved [20, 21]. Based on De Luca [22], the use of electromyography (EMG) is appropriate when assessing neuromuscular pattern during dynamic contraction and assessment of root mean square (RMS) value will better represent signal power. As confirmed by Barbero et al. [23], changes in RMS will indicate that a physiological quantity is changing in time and affecting the signal amplitude.

Recently Nairn et al. [24] defined the instability devices in bottom-up (e.g. balance boards, Swiss or BOSU ball) and top-down (e.g. water pipe) based on the location where instability is applied [25]. Among top-down devices, water-filled pipes have received growing attention, since the stochastic behavior of the water inside the pipe (unstable mass) can produce sudden demands on increased coordination, stabilization patterns and muscle activation [26]. This type of device has been used to investigate kinematics [24, 25] and muscular activity [25–28] of the upper and lower limb muscles during exercising.

However, there is a lack of information regarding neuromuscular patterns of the shoulder dynamic stabilizers during specific shoulder exercises while employing a weight with unstable mass (e.g. a water-filled pipe). Successful reports with reference to such stochastic-behaving weight and its effects on shoulder muscle activation can be extrapolated from recent investigations. In one study [24] the EMG-activity of the prime (m. pectoralis major, triceps brachii, anterior deltoid) and secondary (Mm. latissimus dorsi, biceps brachii, lateral deltoid, upper trapezius) upper-limb movers were assessed during a bench press exercise using a water-filled pipe. The results showed an increased activity of the secondary stabilizer muscles. In another study [28], the neuromuscular activation of the anterior deltoid muscle in trained weightlifters was higher during an overhead squat exercise, when the water in the employed training-tube was free to move along the entire length. Based on the results of these studies, it is reasonable to assume that a weight with a stochastically behaving mass will elicit a greater muscular activation of the shoulder stabilizers when performing specific shoulder exercises. However, it should be defined whether the same amount of total weight combined with a device having an unstable mass behavior (water pipe), will elicit greater EMG-amplitudes of the shoulder stabilizers. Moreover, it is of particular interest to know how the scapular stabilizer ratio is affected when performing a dynamic exercise with a stochastic weight mass. The scapular ratio represents the continuous relationship between the scapula and the humerus, with respect to changing position during dynamic overhead movements [29]. It was reported that an impaired scapular movement or dyskinesia as a result of altered neuromuscular behavior could be the cause of shoulder injuries [30]. It is therefore valuable information for overhead athletes to clarify if an increased instability of weight mass during shoulder exercises will positively affect the activation ratio of the scapula stabilizers, and correspondingly improve injury prevention efficiency.

Therefore, the aim of this study was to investigate the muscle activity of the main shoulder dynamic stabilizers, during three exercises representative for overhead athletes, in four different conditions of weight with stable and unstable mass behavior. It was hypothesized that a combined condition of weight with stable und unstable mass would elicit higher neuromuscular activity than the condition of reduced weight with stable or unstable mass alone.

## Methods

### Participants

Sixteen asymptomatic individuals (7 m/9 f; 28 ± 5 years; 69 ± 11 kg; 174 ± 9 cm) were recruited in a university setting and included in the study after fulfilling inclusion/exclusion criteria. Inclusion criteria were: age (≥18 to 45 years) and weekly sport activity (≥ 3 sport-session/week). Only sport activities demanding a large involvement of the shoulder were included. Exclusion criteria were: pregnancy, infection, cancer, neurological or metabolic diseases, previous shoulder injury (< 2 months) or surgery (< 6 months), pain (shoulder and general) and not-overhead sports (e.g. running, cycling). Before measurements, all participants were clearly (verbally and in written form) informed about all procedure details and signed a written informed consent. The institutional research committee approved the study, according to the Declaration of Helsinki and its amendment in 2008.

### Measurement protocol

First, anthropometrical data, sport activity and training sessions per week were collected using a self-developed questionnaire (Additional file 1). History of injuries, level of mobility, strength and actual pain of the dominant shoulder were investigated using a paper version of the Costant&Murley test [31, 32]. Each participant subjectively rated their current shoulder pain (dominant arm) using a visual analog scale (VAS; 0 = no pain to 10 = maximum imaginable pain). Pain threshold for study inclusion was set at ≤1 (no pain or very mild). Shoulder strength was tested at the end of the questionnaire, consisting of a short functional test [31, 32] that checked if participants were able to hold a 4.5 kg weight in shoulder abduction 90° in the scapular plane with the arm extended for 5-s. This was followed by preparation of the dominant shoulder for electromyographic (EMG) assessment. In detail, six pairs of EMG-electrodes were positioned over six main shoulder muscles as reported in Table 1.

**Table 1:** Definition of the 6 muscles and surface electrode localizations, using an inter-electrode distance of 2 cm

Muscle	Electrode placement
Upper trapezius (U.TA)	Supero-medial and infero-lateral to a point 2 cm lateral to one-half the distance between the C7 spinous process and the lateral tip of the acromion [33]
Lower trapezius (L.TA)	1/3 between the spinous process of the seventh thoracic vertebrae and the medial border of the scapula at the intersection of the scapula spine [34]
Serratus anterior (SA)	Over the seventh intercostal space, just anterior to the fibers of the latissimus dorsi [34]
Lateral deltoid (DE)	Intersection of the midpoint between the anterior and posterior deltoid muscles and the midpoint between the acromion and deltoid tuberosity [33]
Latissimus dorsi (LD)	Posterior axillary fold, directly lateral to the inferior tip of the scapula [33]
Pectoralis major (PE)	3.5 cm medial to the anterior axillary line [33]

Next, a standardized shoulder warm-up and a maximal isometric voluntary contraction (MVIC) were performed by all individuals. MVICs were performed on an isokinetic dynamometer (Contrex MJ; Physiomed Elektromedizin AG, Germany) for the purpose of EMG-normalization. Participants laid supine on the device’s backrest holding a handle with the hand in a supinated position. The trunk was fixed with an additional diagonal belt. The dominant arm was placed in measurement position by the principal investigator, fixed with an additional brace at the proximal part of the elbow. The first MVIC test position was (i) internal/external rotation shoulder abducted to 90° in the scapula plane, neutral humeral rotation, elbow flexed 90° (0° MVIC angle). After a 1-min rest, this was followed by (ii) frontal flexion/extension arm stretched, 90° flexion in the frontal plane (90° MVIC angle). The employed positions demonstrated optimal shoulder muscles voluntary activation during isometric contraction [30, 33, 35] and EMG-MVIC assessment using an isokinetic device reported high reliability [34]. All individuals initially performed a 5-s isometric contraction attempt (familiarization: sub-max. effort). After a 1-min rest, a 5-s MVIC (maximal effort) contraction was produced whilst EMG-data was recorded. This procedure was applied for each movement direction (internal and external rotation, frontal flexion and extension). Afterwards, each participant performed three dynamic shoulder exercises with the dominant shoulder, randomly ordered in a standing position. Exercises were performed holding different custom-built tubes for a total of four conditions (four pipes). EMG of the shoulder muscles was assessed during all three exercises under all four conditions.

### Exercise protocol

The three performed exercises were selected from previous published studies [3, 13, 14] assessing specific exercise programs for the shoulder: (I) internal/external rotation, shoulder abducted to 90° in the scapula plane, neutral humeral rotation, elbow flexed 90° (In/Ex), (II) abduction/adduction, arm straightened, oriented at 30° of scapula plane (scaption) till 90° arm-elevation in frontal plane (Ab/Ad) and (III) flexion/extension, arm outstretched, following a diagonal pattern (start position counter-lateral hip) till 180° shoulder-extension (F/E). During the movements, the hand was rotated continuously, in every repetition, from prone to neutral position in exercises I and III, as well as vice versa in exercise II. Each exercise consisted of five repetitions with a 30-s recovery pause between conditions and was performed under all four conditions (pipes): empty-pipe (P), water-filled-pipe (PW), weight-pipe (PG) and weight + water-filled-pipe (PWG). Except for the initial condition (P) all other conditions were randomly ordered. Between each exercise, a 1-min rest was performed and the order of the exercises was randomized. Participants were instructed to grab the tube in the middle portion and keep a constant-moderate exercise velocity, ≈3-s for each contraction mode (concentric, eccentric). Individuals stood in front of a mirror for visual feedback. The principal investigator constantly checked proper exercise execution (speed, technique) to correct the participant if necessary. All included participants were able to perform all exercises with proper execution on first attempt.

### Exercise tool & conditions

Four pipes with similar basic characteristics (Table 2) were custom-built in order to represent four different exercise conditions. The empty-pipe (P) consisted of a stable mass of low weight (baseline characteristics). The water-pipe (PW) was filled with 550 ml of normal water, representing a condition of low weight with unstable mass behavior and consequential increased instability, due to the water shaking inside the tube. The weight-pipe (PG) had two additional weight plates (2 kg each + 0.5 kg fastening screws) fixed at the bottom of each side, representing a condition of higher weight of stable mass. Last, the water + weight pipe (PWG) consisted of a 400 ml of water and two additional weight plates (1.25 kg each + 1.1 kg fastening/seal up materials) fixed at the pipe’s sides, representative of a combined condition of unstable (water) and stable (weight plate) high weight mass.

**Table 2:** Self-customized pipes, corresponding weight and weight-mass properties

	**Pipe characteristic**		**Mass**	**Total Weight**
	**P**	empty	stable	0.5 kg
	**PW**	water filled	unstable	1 kg
	**PG**	weight added	stable	4.5 kg
	**PWG**	water + weight	unstable	4.5 kg

All pipes have same baseline characteristics: length 50 cm, diameter 6.3cm, orange PVC plastic

### EMG analysis

Neuromuscular activity of the following shoulder muscles was assessed with a 6-lead surface EMG setup: Mm. upper trapezius (U.TA), lower trapezius (L.TA), lateral deltoid (DE), latissimus dorsi (LD), serratus anterior (SA) and pectoralis major (PE). EMG-data were recorded using a wireless surface capture system (Myon 320, RFTD-32, sampling frequency 4000 Hz, myon AG, Switzerland). Electrode (bipolar pre-gelled Ag/AgCl; Ambu, Medicotest, Denmark, type P-00-S) placement was carefully determined (Table 1). The skin was shaved, slightly roughened with sandpaper, to remove surface epithelial layers and disinfected. Inter-electrode impedance was checked to be < 5 kΩ [23, 36]. Wireless transmitters (m320TXA), forwarding the signal to a central receiver unit (m320RX, bandwidth: 5–500 Hz, butterworth filter 4th order, digitized), were placed at the skin and connected to EMG electrodes by short cables. To include a synchronized trigger signal for start- and end-point detection of each movement direction, an additional telemetric accelerometer was used. After detecting the start- and end-point of the movement cycle, signals were A/D-converted (NI PCI 6229, 250 kS/s, 16-Bit, National Instruments®, Austin, TX, USA) and stored on a personal computer (IMAGO record master, LabView®-based, pfitec, biomedical systems, Endingen, Germany). Post-processing of the EMG and ACC data was done using a customized software solution (IMAGO process master, LabView®-based, pfitec, biomedical systems, Endingen, Germany). The collected EMG-data was visually screened for detection of possible artefacts in each contraction mode (concentric, eccentric) for all 5 repetitions. After that, the signals were rectified and the root mean square [RMS (V/s)] was calculated. Maximal voluntary isometric contraction values (MVIC) were obtained calculating 1 s of the highest activity plateau (out of the 5-s measured), using visual inspection by the same principal investigator, for all EMG data.

### Data analysis

All non-digital data were paper-pencil collected in the questionnaire and case report form (CRF) and later transferred to a digital Excel data-sheet (Microsoft, Redmond, WA, USA, Version 15.18). A plausibility check was performed by screening all data-sets for implausible or extreme values. Abnormal values were recalculated or revised in relation to the hand-written CRF information and raw-data. Raw RMS (V/s) and MVIC-normalized RMS (%MVIC) values were averaged across the 5 repetitions for both contraction modes (concentric, eccentric) in all conditions (pipes). Scapular stabilizer (*SR*) and contraction (*CR*) ratios for the normalized amplitudes were calculated for all exercises and conditions. *SR* were calculated by dividing the RMS (%MVIC) of U.TA with RMS (%MVIC) of L.TA and SA [e.g. RMS (%MVIC)-U.TA/RMS (%MVIC)-L.TA]. *CR* were assessed by dividing the mean RMS (%MVIC) of the concentric to the mean RMS (%MVIC) of the eccentric phase [e.g. In/Ex: RMS (%MVIC)-Ex/RMS (%MVIC)-In]. Statistical analysis was performed using the SPSS software (SPSS 21.0, IBM Corp., Armonk, NY, USA). Normal distribution of the data was tested (Shapiro-Wilk) and descriptively analyzed (mean ± SD). Analysis of differences between conditions (pipes) for each muscle was performed for the RMS (%MVIC) values, contraction ratios (*CR*) and scapula ratio (*SR*) using paired t-tests with α level set at *p* < 0.05 and subsequent manual Bonferroni correction (α = 0.008) to account for multiple testing. Pearson’s correlation coefficient (*r*) was calculated for each muscle and reported as mean value, representing consistency of the measurements protocol between conditions. Differences between RMS means (ΔRMS) and percentage of the difference between conditions were calculated.

## Results

### Internal/external rotation (In/Ex)

**Fig. 1:**
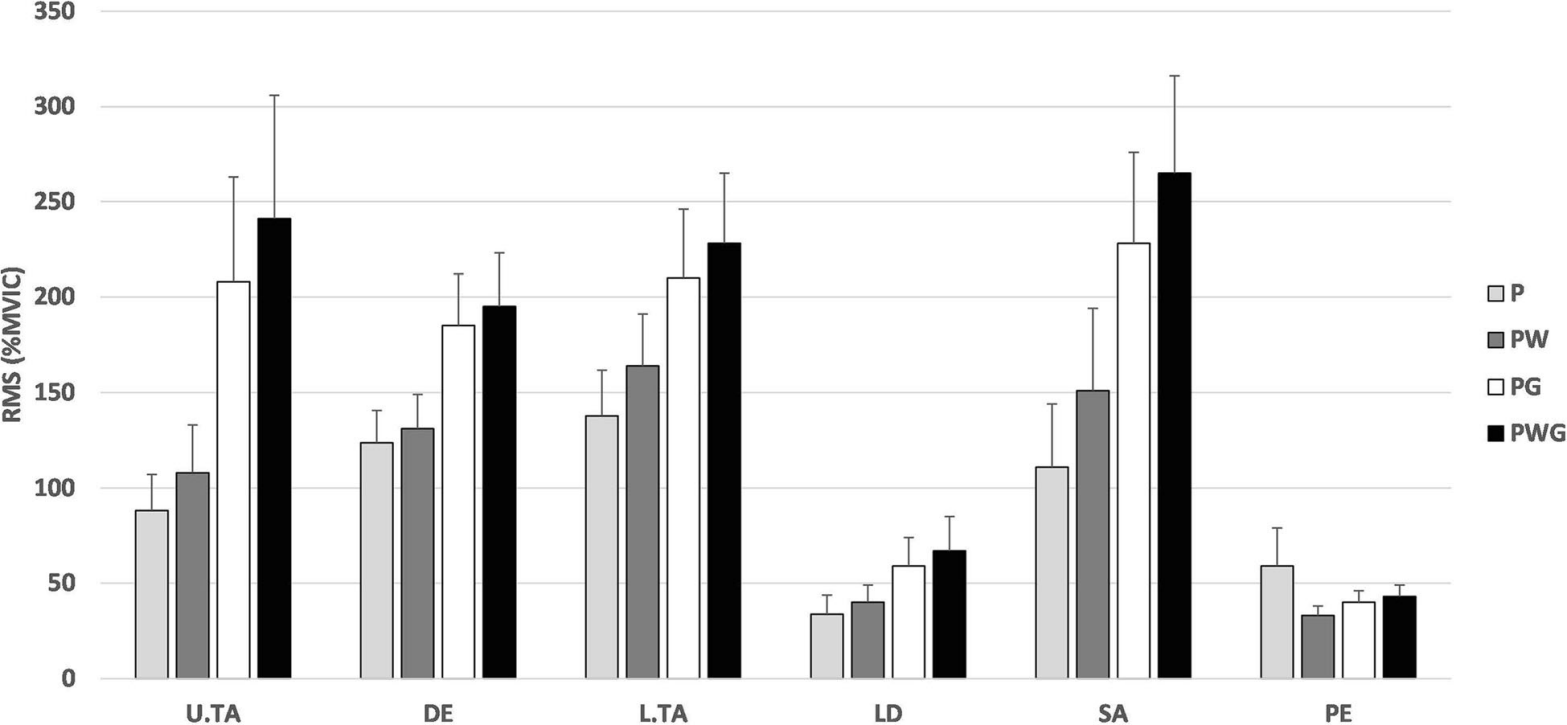
Normalized RMS values for the In/Ex shoulder rotation. Legend: P: empty pipe (0.5 kg); PG: weight (stable mass; 4.5 kg); PW: water (unstable mass; 1 kg); PWG: water + weight (unstable mass; 4.5 kg); U/L .TA: upper/lower trapezius; DE: deltoid; LD: latissimus dorsi; SA: serratus anterior; PE: pectoralis major; RMS (%MVIC): root mean square values normalized to (%MVIC), averaged for the 5 repetitions for both movement directions (mean ± SD)

The normalized (%MVIC) root mean square values for all muscles, in all conditions for shoulder internal/external rotation are displayed in Fig. 1.

The combined condition of weight + water (PWG) reported the highest RMS (%MVIC) values for all muscles, except for the PE muscle. The condition with the stable weighted pipe (PG) reported lower values than PWG, but higher than the baseline-pipe (P) and the water-filled-pipe (PW). The exercise condition P reported the highest value for the PE muscle compared to the other conditions (PW, PG, PWG), but the lowest for all other muscles. Exercising under the PW condition elicited higher muscular activity than P but lower than PG or PWG.

Analysis of differences between RMS (%MVIC) mean muscular activity was significant (*p* < 0.008) between conditions PWG and P, as well as for the unstable condition PW. This was the case for all muscles except for PE in condition P. Comparison between the mean of PG and PWG revealed a statistically significant higher muscle activity in the L.TA and SA muscles only, as displayed in Table 3.

**Table 3:** Comparison of the muscular activity of all six muscles during the In/Ex shoulder rotation in the four conditions (pipes). Displayed are the *p-*values with a statistical difference of *p* < 0.008

Muscle	P vs PG	P vs PW	P vs PWG	PG vs PW	PG vs PWG	PW vs PWG	*r*
**U.TA**	0.010	0.069	0.010	0.006*	0.020	0.006*	0.915
**DE**	0.001*	0.052	< 0.001*	0.001*	0.126	< 0.001*	0.931
**L.TA**	< 0.001*	0.006*	< 0.001*	0.001*	0.005*	< 0.001*	0.956
**LD**	0.002*	0.236	0.005*	0.002*	0.038	0.002*	0.932
**SA**	< 0.001*	0.006*	< 0.001*	< 0.001*	< 0.001*	< 0.001*	0.946
**PE**	0.327	0.202	0.402	0.001*	0.042	0.002*	0.747

P: empty pipe (0.5 kg); PG: weight (stable mass; 4.5 kg); PW: water (unstable mass; 1 kg); PWG: water + weight (unstable mass; 4.5 kg); U/L. TA: upper/lower trapezius; *DE* deltoid, *LD* latissimus dorsi, *SA* serratus anterior, *PE* pectoralis major; *r*: mean correlation coefficient; *: significant differences α level Bonferroni adjusted

### Abduction/adduction (Ab/Ad)

**Fig. 2:**
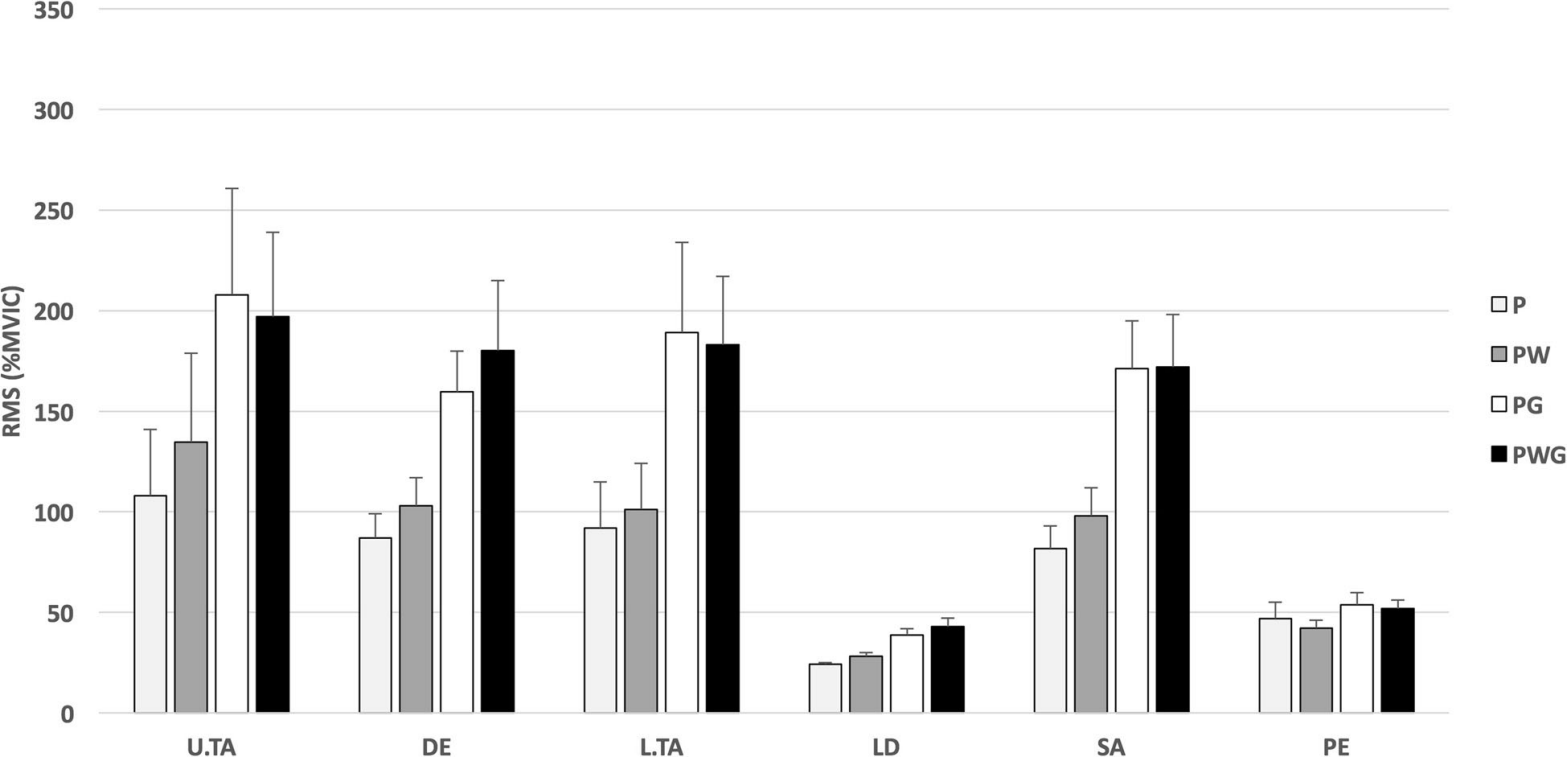
Normalized muscular activity values for the shoulder Ab/Ad. Legend: P: empty pipe (0.5 kg); PG: weight (stable mass; 4.5 kg); PW: water (unstable mass; 1 kg); PWG: water + weight (unstable mass; 4.5 kg); U/L .TA: upper/lower trapezius; DE: deltoid; LD: latissimus dorsi; SA: serratus anterior; PE: pectoralis major; RMS (%MVIC): root mean square values normalized to (%MVIC), averaged for the 5 repetitions for both movement directions (mean ± SD)

The normalized (%MVIC) root mean square values for all muscles, in all conditions for the shoulder abduction/adduction are displayed in Fig. 2.

The conditions PWG and PG reported very similar RMS (%MVIC) values in the DE muscle the main difference of ±20%. Condition P reported the lowest muscular RMS (%MVIC) for all muscles, except for the PE muscle. Exercising under the condition PW elicited amplitudes higher than P, but below the PG and the PWG values.

Analysis of differences between conditions, revealed a statistically significant (*p* < 0.008) difference of the mean between the conditions PWG, P and PW for the DE, L.TA, LD and SA muscles. No significant difference was detected between high stable weight condition (PG) and PWG for all muscles. Muscle SA reported a significant difference between the mean of P and PW as reported in Table 4.

**Table 4:** Comparison of the muscular activity of all six muscles during the shoulder Ab/Ad in the four conditions (pipes). Displayed are the *p-*values with a statistical difference of *p* < 0.008

Muscle	P vs PG	P vs PW	P vs PWG	PG vs PW	PG vs PWG	PW vs PWG	*r*
**U.TA**	< 0.001*	0.043	0.001*	0.002*	0.607	0.017	0.921
**DE**	< 0.001*	0.046	0.001*	< 0.001*	0.337	0.004*	0.885
**L.TA**	0.001*	0.042	< 0.001*	0.002*	0.700	0.001*	0.937
**LD**	< 0.001*	0.019	< 0.001*	0.001*	0.072	< 0.001*	0.873
**SA**	< 0.001*	0.001*	< 0.001*	< 0.001*	0.934	< 0.001*	0.903
**PE**	0.303	0.538	0.320	0.011	0.786	0.006*	0.802

P: empty pipe (0.5 kg); PG: weight (stable mass; 4.5 kg); PW: water (unstable mass; 1 kg); PWG: water + weight (unstable mass; 4.5 kg); U/L. TA: upper/lower trapezius; *DE* deltoid, *LD* latissimus dorsi, *SA* serratus anterior, *PE* pectoralis major; *r*: mean correlation coefficient; *: significant differences α level Bonferroni adjusted

### Diagonal flexion/extension (F/E)

**Fig. 3:**
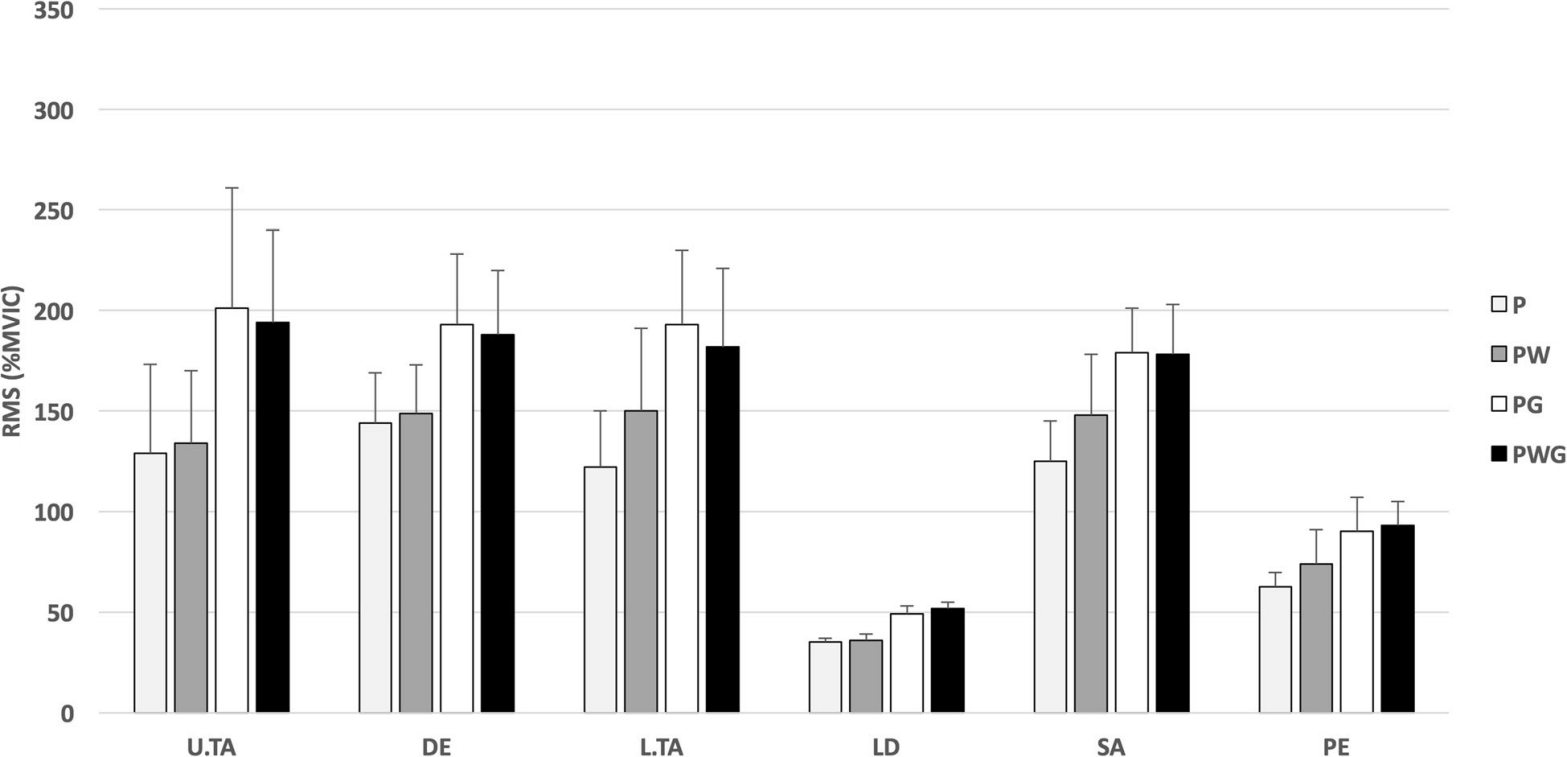
Normalized muscular activity for the diagonal shoulder F/E. Legend: P: empty pipe (0.5 kg); PG: weight (stable mass; 4.5 kg); PW: water (unstable mass; 1 kg); PWG: water + weight (unstable mass; 4.5 kg); U/L .TA: upper/lower trapezius; DE: deltoid; LD: latissimus dorsi; SA: serratus anterior; PE: pectoralis major; RMS (%MVIC): root mean square values normalized to (%MVIC), averaged for the 5 repetitions for both movement directions (mean ± SD)

The normalized amplitudes (%MVIC) for all muscles, in all conditions for the diagonal shoulder flexion/extension exercise are displayed in Fig. 3.

The exercise performed under condition PG reported the highest RMS (%MVIC) for muscles U.TA, DE, L.TA and SA. Values of the LD and PE muscles in condition PG, were lower than in condition PWG, but higher than those in P and PW. Exercising under condition P elicited in all muscles the lowest RMS (%MVIC). The condition PW reported higher activation than P in all muscles but lower than in conditions PG and PWG.

Statistically significant (*p* < 0.008) differences were found between conditions PWG and P in all observed muscles except in the DE muscle. Between PG and P mean differences were statistically significant for all muscles, except for the DE. No significant differences were found comparing PW and P conditions, as well as between the PG and PWG, as displayed in Table 5.

**Table 5:** Comparison of the muscular activity of all six muscles during the diagonal shoulder F/E in the four conditions (pipes). Displayed are the *p-*values with a statistical difference of *p* < 0.008

Muscle	P vs PG	P vs PW	P vs PWG	PG vs PW	PG vs PWG	PW vs PWG	*r*
**U.TA**	0.003*	0.794	0.003*	0.020	0.666	0.002*	0.950
**DE**	0.011*	0.745	0.017	0.027	0.740	0.066	0.879
**L.TA**	0.001*	0.120	0.004*	0.012	0.228	0.067	0.934
**LD**	< 0.001*	0.529	< 0.001*	< 0.001*	0.302	< 0.001*	0.769
**SA**	< 0.001*	0.130	0.001*	0.113	0.905	0.160	0.864
**PE**	0.033	0.326	0.001*	0.006*	0.845	0.234	0.747

P: empty pipe (0.5 kg); PG: weight (stable mass; 4.5 kg); PW: water (unstable mass; 1 kg); PWG: water + weight (unstable mass; 4.5 kg); U/L. TA: upper/lower trapezius; *DE* deltoid, *LD* latissimus dorsi, *SA* serratus anterior, *PE* pectoralis major; *r*: mean correlation coefficient; *: significant differences α level Bonferroni adjusted

### Contraction ratio (*CR*), scapula stabilizer ratio (*SR*), raw RMS (V/s), differences of the means (ΔRMS)

**Fig. 4:**
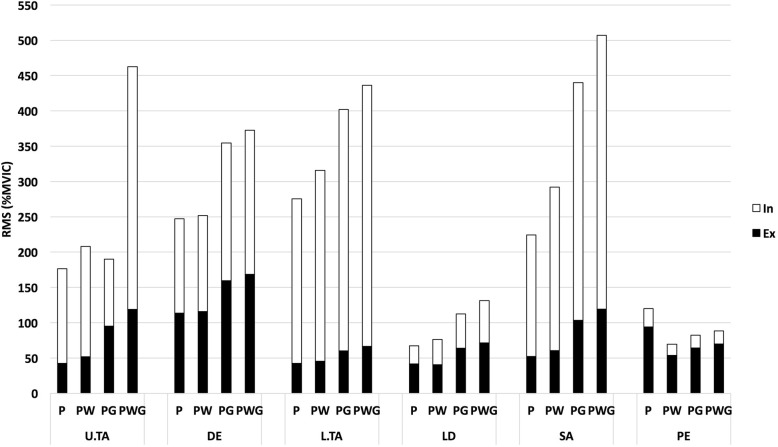
Contraction ratio for the In/Ex shoulder rotation. Legend: P: empty pipe (0.5 kg); PG: weight (stable mass; 4.5 kg); PW: water (unstable mass; 1 kg); PWG: water + weight (unstable mass; 4.5 kg); U/L .TA: upper/lower trapezius; DE: deltoid; LD: latissimus dorsi; SA: serratus anterior; PE: pectoralis major; CON: concentric; ECC: eccentric; RMS (%MVIC): root mean square values normalized to (%MVIC), averaged for the 5 repetitions for both movement directions (mean ± SD)

**Fig. 5:**
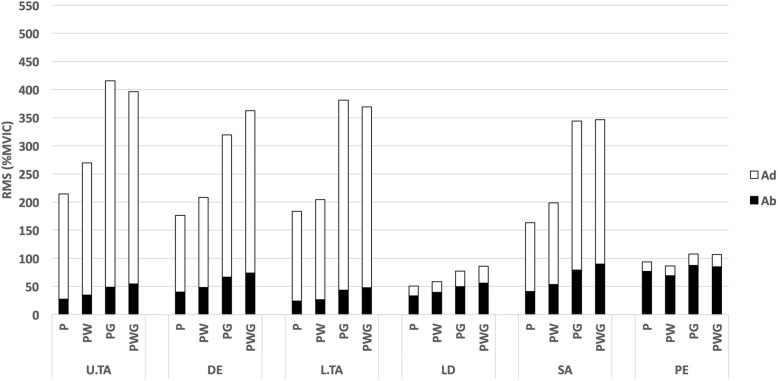
Contraction ratio for the shoulder Ad/Ab. Legend: P: empty pipe (0.5 kg); PG: weight (stable mass; 4.5 kg); PW: water (unstable mass; 1 kg); PWG: water + weight (unstable mass; 4.5 kg); U/L .TA: upper/lower trapezius; DE: deltoid; LD: latissimus dorsi; SA: serratus anterior; PE: pectoralis major; CON: concentric; ECC: eccentric; RMS (%MVIC): root mean square values normalized to (%MVIC), averaged for the 5 repetitions for both movement directions (mean ± SD)

**Fig. 6:**
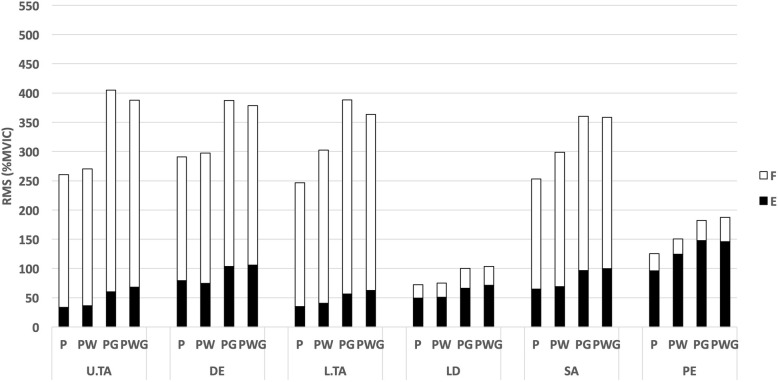
Contraction ratio for the diagonal shoulder F/E. Legend: P: empty pipe (0.5 kg); PG: weight (stable mass; 4.5 kg); PW: water (unstable mass; 1 kg); PWG: water + weight (unstable mass; 4.5 kg); U/L .TA: upper/lower trapezius; DE: deltoid; LD: latissimus dorsi; SA: serratus anterior; PE: pectoralis major; CON: concentric; ECC: eccentric; RMS (%MVIC): root mean square values normalized to (%MVIC), averaged for the 5 repetitions for both movement directions (mean ± SD)

The calculated differences between the mean of each condition (∆RMS) and the relative difference of the percentage (% dif) are reported on Table 6. Computed scapula stabilizer ratio (*SR*) of the three performed exercises during all four conditions are displayed in Table 7. Analysis of differences revealed a statistically significant difference only for the *SR* ratio U.TA/L.TA between the baseline condition (P) and the lower unstable weight condition (PW) compared to the combined condition (PWG). RMS (%MVIC) values of both dynamic phases (concentric, eccentric) for all three exercises are displayed in Figures 4, 5 and 6 in the Appendix. Overall, exercising under the combined condition (PWG) elicited the highest raw RMS (V/s) values in all muscles in all three exercises, except for PE in the internal/external rotation and abduction/adduction exercises. Performing under the stable weight condition (PG) showed RMS (V/s) values lower or similar to PWG in all exercises. The baseline condition (P) registered the lowest neuromuscular activation (V/s) in all muscles for all exercises with the exception of the PE muscle in the In/Ex rotation. The lower unstable weight condition (PW) elicited higher muscular activation compared with condition P, but rather lower than in conditions PG and PWG. The raw EMG activity (RMS V/s) of all muscles for all performed exercises is displayed in Table 8 in the Additional file 2. Mean movement-direction time (concentric, eccentric) for all repetitions was 2.6 ± 0.5 s. in all exercises, which was in line with the requested constant-moderate exercise velocity. The contraction ratio (*CR*) values for the three observed exercises, for all muscles and for all conditions are reported in Table 9 in the Additional file 3. Differences between the *CR* ratio values were not significant (*p* > 0.008) for all muscles in all conditions.

**Table 6:** Calculated RMS (%MVIC) differences between the mean (∆RMS) and percentage of the differences (% dif) between conditions for all performed exercises and investigated muscles

		Conditions											
Exercise	Muscle	P vs PG		P vs PW		P vs PWG		PG vs PW		PG vs PWG		PW vs PWG	
		∆RMS	% dif	∆RMS	% dif	∆RMS	% dif	∆RMS	% dif	∆RMS	% dif	∆RMS	% dif
**In/Ex rotation**	**U.TA**	120	81.1	20	20.3	153	92.1	100*	63.3	33	14.7	133*	76.1
	**DE**	61*	39.5	7	5.5	71*	44.4	54*	34.2	10	5.3	64*	39.3
	**L.TA**	72*	41.4	26*	17.1	90*	49.2	46*	24.6	18*	8.1	64*	32.5
	**LD**	25*	53.8	6	16.1	33*	65.2	19*	38.4	8	12.7	27*	50.5
	**SA**	117*	68.9	40*	30.4	154*	81.8	77*	40.5	37*	15	114*	54.7
	**PE**	19	38.4	26	56.4	16	31.4	7*	19.2	3	7.1	10*	26.2
**Ab/Ad**	**U.TA**	100*	63.3	27	22.1	89*	58.4	73*	42.6	11	5.3	62	37.2
	**DE**	73*	58.9	16	16.7	93*	69.6	57*	43.2	20	11.6	77*	54.3
	**L.TA**	97*	62.9	9	9.2	91*	66.2	88*	60.7	6	3.1	82*	57.6
	**LD**	15*	47.5	4	15.4	19*	56.7	11*	32.7	4	9.6	15*	42.1
	**SA**	89*	70.3	16*	17.8	90*	70.7	73*	54.3	1	0.6	74*	54.7
	**PE**	7	13.9	5	11.1	5	10.0	12	25	2	3.8	10*	22.9
**diagonal F/E**	**U.TA**	72*	43.5	5	3.7	65*	40.1	67	40.0	7	3.4	60*	36.6
	**DE**	49*	28.9	5	3.3	44	26.4	44	25.6	5	2.5	39	23
	**L.TA**	71*	45.1	28	20.6	60*	39.5	43	25.1	11	5.7	32	19.3
	**LD**	14*	33.3	1	2.7	17*	39.1	13*	30.6	3	5.8	16*	36.3
	**SA**	54*	35.4	23	16.7	53*	35	31	18.9	1	0.6	30	18.3
	**PE**	27	35.3	11	16.1	30*	38.5	16*	19.4	3	3.3	19	22.7

P: empty pipe (0.5 kg); PG: weight (stable mass; 4.5 kg); PW: water (unstable mass; 1 kg); PWG: water + weight (unstable mass; 4.5 kg); U/L. TA: upper/lower trapezius; *DE* deltoid, *LD* latissimus dorsi, *SA* serratus anterior, *PE* pectoralis major; *: statistical significant difference *p* < 0.008

**Table 7:** Calculated scapula stabilizer ratio (*SR*) for the muscle U.TA, L.TA and SA for all performed exercises (mean ± SD) in all the four conditions (pipes)

	Intra/Extra rotation (In/Ex)		Abduction/Adduction (Ab/Ad)		Flexion/Extension diagonal (F/E)	
	U.TA/L.TA	U.TA/SA	U.TA/L.TA	U.TA/SA	U.TA/L.TA	U.TA/SA
**P**	0.65 ± 0.52 ^**a**^	1.20 ± 1.25	1.32 ± 2.04	1.35 ± 1.36	0.94 ± 0.80	0.84 ± 0.67
**PW**	0.68 ± 0.50 ^**b**^	1.09 ± 1.20	1.35 ± 1.77	1.36 ± 1.40	0.86 ± 0.60	0.80 ± 0.67
**PG**	0.98 ± 0.86	1.10 ± 1.03	1.03 ± 0.73	1.21 ± 1.06	0.82 ± 0.46	0.90 ± 0.80
**PWG**	1.08 ± 0.86 ^**a, b**^	1.07 ± 1.13	1.03 ± 0.73	1.31 ± 1.20	0.97 ± 0.56	0.89 ± 0.66

P: empty pipe (0.5 kg); PG: weight (stable mass; 4.5 kg); PW: water (unstable mass; 1 kg); PWG: water + weight (unstable mass; 4.5 kg); U/L. TA: upper/lower trapezius; *SA* serratus anterior; Scapula stabilizer ratio: U.TA RMS (%MVIC)/L.TA RMS (%MVIC); U.TA RMS (%MVIC)/SA RMS (%MVIC); a,b: significant difference (*p* < 0.008)

## Discussion

The aim of the study was to investigate the neuromuscular activity of the dynamic stabilizers in the shoulder, when performing three shoulder exercises under four different conditions (P, PG, PW, PWG). It was assumed that a combined condition of stable and stochastic weight mass (PWG), would elicit higher levels of muscular activity. The results support this assumption, particularly for the internal/external rotation exercise (In/Ex), where condition PWG showed significantly higher RMS (%MVIC) for the lower trapezius (L.TA) and for the serratus anterior (SA) compared to all other conditions. Despite PWG eliciting higher RMS (%MVIC) mean values for the abduction/adduction (Ab/Ad) as well as for the flexion/extension (F/E) exercises, the results were not statistically significant when compared to performing with the stable weight mass (PG). Analysis of Pearson’s correlation resulted in a strong correlation (e.g. mean *r* > 0.747) between conditions in all muscles, representing the validity of the measurement protocol and the linear correlation in each subject between measurement conditions (pipes).

For all three exercises, a similar relationship between increment in total weight and corresponding level of muscular activity was observed. Taking into account the rise in total weight used during the exercise (P: 0.5 kg, PW: 1 kg, PG, PWG: 4.5 kg) a steady increase in the muscular activity (RMS %MVIC) is observable, following the principle of low weight = lower activation to higher weight = higher activation. This could be explained by an increase in external resistance, a corresponding increase in muscle fiber recruitment [37] and a consequential elevation in neuromuscular activation [38, 39]; causing the incrementing EMG activity between conditions P, PW and PG, PWG in the observed exercises. Furthermore, for the same amount of weight a stochastic distribution (e.g. PWG) will elicit an additional increment of the EMG-muscle activity.

In this case, a gradual progression from condition P to condition PWG (P → PW → PG → PWG) is depicted, corresponding to a progression from a lower to higher demand training condition. As discussed in section 2.4, in conditions PW and PWG the total weight partly consists of an unstable chaotically-behaving mass (water) that reproduces a consequential augmented degree of freedom in the resistance load applied during the dynamic exercise [24]. Specifically, the water-filled pipes generate a different weight distribution during exercising along the length, due to the unpredictable movements of the water inside the tube [27, 28]. This unpredictable mass distribution is independent from the exercise performed, thus the new force distribution creates unstable conditions where the muscles need to compensate in order to hold the tube steady while exercising [26]. Consequently, pipes PW and PWG have a specific unstable-dynamic-behavior that can be defined as an unstable condition. Due to this, the stabilization system is challenged to compensate for the unpredictable force distribution occurring while dynamically exercising with the water-filled pipe [28]. This was significantly evident for internal/external rotation (In/Ex), but not for the abduction/adduction (Ab/Ad) and diagonal flexion/extension (F/E) exercises. An explanation for this could be the differences in arm positioning. In the observed In/Ex rotation on 90° of shoulder abduction in the scapular plane, the arm was flexed. In contrast, during Ab/Ad and diagonal F/E, the arm was completely extended, possibly resulting in increased forearm muscle activity. Another factor could be a faulty execution of the dynamic movement, which did not generate enough water displacement inside the tube. In order to generate enough water displacement, all subjects were instructed to additionally rotate their hands while performing the exercises. During In/Ex rotation the hand started in prone position and was turned in half supination (hammer position) when the shoulder was in complete external rotation. For the diagonal F/E exercise, the same protocol was applied, starting with a pronated hand and encouraging a half-pronated position at 180° shoulder extension. During shoulder abduction/adduction, the hand was turned in the opposite sequence, starting from half-pronated position to pronated position at 90° abduction. It is likely that the water displacement generated in the In/Ex shoulder rotation was superior to the two other exercises, resulting in the higher neuromuscular activity (RMS %MVIC) observed.

When considering only the upper/lower trapezius and the serratus muscles, the observed neuromuscular pattern and their consequent ratio (*SR*) varied among the three performed exercises, in line with current literature [40, 41]. In particular, it has been reported that an excessive compensatory muscular activation of the upper trapezius (U.TA), combined with a decrease of the lower trapezius (L.TA) and serratus (SA) activity contribute to impaired scapular function during dynamic movements [30]. Such an impaired ratio was identified as a potential risk factor for shoulder injuries. The optimal ratio has been considered to be ≤1.00, which corresponds to a lower U.TA compensatory activation [30].

In internal/external shoulder rotation, exercising under condition PG and PWG showed a significant increase in the *SR* ratio between U.TA and L.TA, as compared to P and PW. This could be caused by the higher total weight used, with a consequential increased muscular activation of the upper trapezius. This might be particularly noteworthy in the eccentric dynamic phase in order to decelerate the weight, stop the movement direction and then start the concentric phase. The abduction/adduction exercise showed an inverted pattern. With lower weight (P, PW), ratios were found > 1.00. When the weight was raised, independently of the stable (PG) or unstable (PWG) weight mass, the values for the U.TA/L.TA were closer to 1.00, similarly to a previous investigation [42]. An additional explanation could be the different lever generated in In/Ex rotation versus Ab/Ad due to the different arm position. However, in both exercises the ratio between U.TA and SA muscles showed the same values (> 1.00). For the diagonal F/E exercise the *SR* ratios (U.TA/L.TA, U.TA/SA) remained < 1.00 in all conditions. Accordingly, it may be concluded that performing shoulder abduction/adduction with higher weight will increase lower trapezius activation and internal/external rotation will decrease lower trapezius activation. This occurred regardless of the stable or unstable nature of the weight mass employed during the dynamic movements. Regarding the relationship between the RMS (%MVIC) in the concentric and the eccentric phase of the movement of each exercise, the instability of the weight mass had no influence on contraction ratios (*CR*).

Another important consideration for clinicians regarding the interaction of the total weight and weight mass properties (stable or unstable) is the effect on the shoulders’ neuromuscular pattern. During the internal/external rotation exercise, performing with low weight conditions (P, PW) resulted in a different model of muscular activation than performing under higher weight conditions (PG, PWG). Based on these results, training with a higher weight will elicit an increased demand of the muscles controlling scapula displacement, increasing the risk of possible alteration in stabilizers co-activation. Exercising with lower weight and with a stochastic weight mass (PW), elicited a significantly higher activation of the observed muscle compared to only a lower weight stable mass (P). However, this difference was only significant for the L.TA. In contrast, with higher weight and unstable mass (PWG), statistically significant differences were observed in the U.TA, L.TA and SA. It can be concluded that training with an unstable weight mass and a higher amount of total weight will enhance muscle activity of the shoulder stabilizers. This is valuable information for clinicians in order to tailor exercise protocols to specific patient or athlete needs. It should be noted however, that this effect was particularly evident in the In/Ex rotation exercise. Statistically significant differences were detected in the abduction/adduction exercise only between the condition P and PW for SA and LD muscles. For the diagonal flexion/extension (F/E), the neuromuscular pattern remained the same independent of total weight employed or instability of the weight mass.

## Limitations

A few methodological limitations are present in this study and should be carefully reviewed. During the EMG analysis we used a standardized MVIC measurement performed on an isokinetic device for all muscles instead of the employment of several single muscle-functional tests. This has the advantage to highly standardize the test procedure between participants and reduce potential rater-effects. However, due to test positions and isometric testing on an isokinetic device, it cannot ruled out that single muscles were not activated to their highest level [43]. This may explain the high percentages (e.g. SA: 254 ± 51%MVIC) when the raw EMG-RMS (V/s) values were normalized to the MVIC-RMS (V/s). However, such RMS (%MVIC) are consistent with the literature when an EMG-signal of a dynamic movement is normalized to an isometric contraction [43, 44]. For future research, the employment of kinematic analysis is recommended in order to evaluate participants’ control strategies and determine weight displacement between conditions (pipes) during exercise. Additionally, the amount of weight used was the same for all subjects, since it was not feasible to adjust weight to each participant due to the self-customized nature of the pipes. Furthermore, due to limitation in the customization, weight difference between pipes P and PW (500 g) was impossible to overcome preserving pipe’s handling property.

To ensure that all individuals could exercise with the 4 kg pipes, a strength test as described in the measurement protocol, was performed. The total weight used in this study is in accordance with a previous study on a cohort of the same age [41]. However, it cannot be assumed that the same exercise intensity was performed between subjects. Finally, only a sample of asymptomatic trained people was investigated. This limits the transfer of the findings to a symptomatic population.

## Conclusion

The results demonstrated that training with an unstable weight mass (PW, PWG) elicited greater muscle activity in the shoulder muscles when compared to a weight with stable mass behavior (P, PG). The observed increase in neuromuscular activity could be attributed to the increased stabilization demands induced by the unstable mass. Particularly, in the internal/external shoulder rotation (In/Ex) exercise, the pipe PWG showed significantly greater muscle activity for the lower trapezius and serratus anterior when compared to the other conditions (P, PW, PG). A differentiation in the neuromuscular pattern, displaying an increased activity of the muscles involved in shoulder stabilization, was evident for the In/Ex rotation between the conditions P, PW and PG, PWG. Performing exercises with a stochastic-behavior weight did not change the contraction ratio (*CR*) between concentric and eccentric phases of the dynamic movement. Furthermore, the ratio of the muscle acting on scapular stabilization muscles (*SR*) such as lower/upper trapezius (U.TA/L.TA) and serratus (SA) was significantly affected by the instability of weight mass only in the In/Ex shoulder rotation. Physio- and Sports- therapists can refer to the results of this study when implementing exercise programs for shoulder injury prevention. Additionally, the use of a stochastic-behavior mass could increase exercise efficiency during performance. However, the reader should keep in mind that such findings were observed in a healthy trained cohort and functional benefits remain unclear in different populations. We recommend therefore that future randomized controlled trials should investigate such aspects in different specific cohorts like elderly or young athletes.

### Supplementary information

**Additional file 1.** Participant Questionnaire.

**Additional file 2: Table 8.** Raw RMS (V/s) as mean values of the five repetitions for the two phases of the movement (concentric, eccentric) of each investigated exercise (mean ± SD) in all the four conditions.

**Additional file 3: Table 9.** Calculated contraction ratio values (*CR*) between movement mode (concentric/eccentric) for all performed exercises and observed muscles (mean ± SD) in the four conditions (pipes).

## Data Availability

All data generated or analyzed during this study are included in this published article and its supplementary information files.

## Abbreviations

% dif: Percentage difference
∆RMS: Difference between the means
Ab/Ad: Abduction/Adduction
*CR*: Contraction ratio
DE: M. Lateral Deltoid
F/E: Flexion/Extension
In/Ex: Internal /External rotation
L.TA: M. Lower Trapezius
LD: M. Latissimus Dorsi
MVIC: Maximal Voluntary Isometric Contraction
P: Empty pipe
PE: M. pectoralis Major
PG: Additional weight pipe
PW: Water filled pipe
PWG: Water + additional weight pipe
RMS (%MVIC): Root Mean Square normalized to MVIC
RMS: Root Mean Square
SA: M. serratus Anterior
*SR*: Scapular ratio
U.TA: M. Upper Trapezius
