# Supplementary material for: Neuromuscular shoulder activity during exercises with different combinations of stable and unstable weight mass
Source: BMC Sports Sci Med Rehabil. 2020 Mar 26;12:21. doi: 10.1186/s13102-020-00168-x (PMC7098120; doi:10.1186/s13102-020-00168-x)
Supplement: Supplementary file 1 — Additional file 1. Participant Questionnaire. [file 13102_2020_168_MOESM1_ESM.docx]

**Master Shoulder**

**EMG-Measurement/Instability weight mass**

**Participant Questionnaire**

Universität Potsdam - Hochschulambulanz

Zentrum für Sportmedizin

Am Neuen Palais 10

Haus 12

14469 Potsdam

Researcher: Omar Baritello

First Supervisor: Dr. Steffen Müller

Second Supervisor: Dr. Tilman Engel

**Participant questionnaire**

**Part I**

**Anthropometrics - Sport activity**

Universität Potsdam - Hochschulambulanz

Zentrum für Sportmedizin

Am Neuen Palais 10

Haus 12

14469 Potsdam

Researcher: Omar Baritello

First Supervisor: Dr. Steffen Müller

Second Supervisor: Dr. Tilman Engel

Assistent:

**a) Anthropometrics:**

*Date: ________________ *Sex: □ m □ w

*Height: _______ cm *Weight: ______ Kg

*Dominat shoulder: □ Right □ Left

| **b) Wich sport activity do you perform regularly every week?** | | |
| --- | --- | --- |
| **A**  .......................................  (Sport activity name) | **B**  .......................................  (Sport activity name) | **C**  .......................................  (Sport activity name) |
| I performed activity **A** in the **last 4 Weeks**  .......... **Times** (Sessions),  every **Session lasted:**  For ca. ......... **Hours**  or  ca. ............ **Minutes** | I performed activity **B** in the **last 4 Weeks**  .......... **Times** (Sessions),,  every **Session lasted:**  For ca. ......... **Hours**  or  ca. ............ **Minutes** | I performed activity **C** in the **last 4 Weeks**  .......... **Times** (Sessions),,  every **Session lasted:**  For ca. ......... **Hours**  or  ca. ............ **Minutes** |
| Professional Athlete category:_________ | Professional Athlete category:_________ | Professional Athlete category:_________ |

Comments:

__________________________________________________________________________________________________________________________________________
