# Supplementary material for: Neuromuscular shoulder activity during exercises with different combinations of stable and unstable weight mass
Source: BMC Sports Sci Med Rehabil. 2020 Mar 26;12:21. doi: 10.1186/s13102-020-00168-x (PMC7098120; doi:10.1186/s13102-020-00168-x)
Supplement: Supplementary file 2 — Additional file 2: Table 8. Raw RMS (V/s) as mean values of the five repetitions for the two phases of the movement (concentric, eccentric) of each investigated exercise (mean ± SD) in all the four conditions. [file 13102_2020_168_MOESM2_ESM.docx]

**Appendix 1. (Supplementary material)**

| **Table 8.**  Raw RMS (V/s) as mean values of the five repetitions for the two phases of the movement (concentric, eccentric) of each investigated exercise (mean±SD) in all the four conditions. | | | | | | | | |
| --- | --- | --- | --- | --- | --- | --- | --- | --- |
| **Exercise** | **Condition** | | **U.TA** | **DE** | **L.TA** | **LD** | **SA** | **PE** |
| **Intra/Extra**  **rotation (In/Ex)** | **P** | IN | 0.09±0.06 | 0.20±0.07 | 0.20±0.12 | 0.04±0.02 | 0.16±0.10 | 0.05±0.06 |
|  |  | EX | 0.10±0.06 | 0.18±0.08 | 0.22±0.15 | 0.05±0.03 | 0.20±0.15 | 0.04±0.04 |
|  | **PW** | IN | 0.11±0.06 | 0.20±0.07 | 0.24±0.14 | 0.05±0.03 | 0.22±0.15 | 0.03±0.01 |
|  |  | EX | 0.12±0.07 | 0.20±0.08 | 0.24±0.13 | 0.05±0.03 | 0.23±0.14 | 0.03±0.01 |
|  | **PG** | IN | 0.20±0.11 | 0.28±0.09 | 0.30±0.18 | 0.07±0.05 | 0.34±0.21 | 0.03±0.01 |
|  |  | EX | 0.21±0.12 | 0.27±0.09 | 0.32±0.18 | 0.08±0.05 | 0.38±0.21 | 0.03±0.01 |
|  | **PWG** | IN | 0.24±0.13 | 0.29±0.11 | 0.33±0.18 | 0.08±0.05 | 0.40±0.23 | 0.03±0.01 |
|  |  | EX | 0.26±0.14 | 0.29±0.10 | 0.35±0.18 | 0.09±0.06 | 0.44±0.28 | 0.03±0.01 |
| **Abduction/Adduction**  **(Ab/Ad)** | **P** | AB | 0.12±0.11 | 0.19±0.15 | 0.14±0.12 | 0.05±0.02 | 0.18±0.11 | 0.04±0.02 |
|  |  | AD | 0.10±0.09 | 0.14±0.07 | 0.12±0.10 | 0.04±0.02 | 0.14±0.09 | 0.04±0.03 |
|  | **PW** | AB | 0.16±0.14 | 0.23±0.16 | 0.15±0.11 | 0.05±0.03 | 0.22±0.15 | 0.03±0.02 |
|  |  | AD | 0.12±0.10 | 0.17±0.09 | 0.13±0.09 | 0.04±0.02 | 0.17±0.11 | 0.03±0.02 |
|  | **PG** | AB | 0.22±0.15 | 0.30±0.20 | 0.24±0.14 | 0.07±0.05 | 0.31±0.22 | 0.04±0.02 |
|  |  | AD | 0.21±0.13 | 0.26±0.11 | 0.24±0.18 | 0.07±0.03 | 0.30±0.16 | 0.04±0.02 |
|  | **PWG** | AB | 0.25±0.13 | 0.32±0.13 | 0.25±0.16 | 0.07±0.04 | 0.35±0.19 | 0.04±0.01 |
|  |  | AD | 0.22±0.14 | 0.29±0.21 | 0.24±0.16 | 0.07±0.05 | 0.31±0.23 | 0.04±0.02 |
| **Flexion/Extension**  **(F/E)** | **P** | F | 0.13±0.08 | 0.22±0.11 | 0.16±0.15 | 0.05±0.04 | 0.23±0.21 | 0.06±0.02 |
|  |  | E | 0.14±0.10 | 0.28±0.13 | 0.20±0.11 | 0.07±0.02 | 0.31±0.14 | 0.05±0.07 |
|  | **PW** | F | 0.13±0.11 | 0.20±0.11 | 0.19±0.14 | 0.06±0.04 | 0.26±0.18 | 0.05±0.04 |
|  |  | E | 0.16±0.07 | 0.29±0.09 | 0.22±0.17 | 0.07±0.02 | 0.31±0.18 | 0.06±0.02 |
|  | **PG** | F | 0.19±0.16 | 0.29±0.13 | 0.24±0.17 | 0.08±0.04 | 0.33±0.25 | 0.06±0.04 |
|  |  | E | 0.27±0.13 | 0.36±0.16 | 0.29±0.14 | 0.09±0.05 | 0.41±0.17 | 0.07±0.03 |
|  | **PWG** | F | 0.20±0.20 | 0.27±0.17 | 0.22±0.20 | 0.07±0.05 | 0.32±0.25 | 0.08±0.03 |
|  |  | E | 0.31±0.12 | 0.42±0.12 | 0.33±0.14 | 0.10±0.03 | 0.45±0.16 | 0.07±0.04 |

P: empty pipe (0.5 kg); PG: weight (stable mass; 4.5 kg); PW: water (unstable mass; 1 kg); PWG: water + weight (unstable mass; 4.5 kg); U/L .TA: upper/lower trapezius; DE: deltoid; LD: latissimus dorsi; SA: serratus anterior; PE: pectoralis major; Grey shadow: highest value of EMG-activity, root mean square (RMS) (V/s)
