# Supplementary material for: Neuromuscular shoulder activity during exercises with different combinations of stable and unstable weight mass
Source: BMC Sports Sci Med Rehabil. 2020 Mar 26;12:21. doi: 10.1186/s13102-020-00168-x (PMC7098120; doi:10.1186/s13102-020-00168-x)
Supplement: Supplementary file 3 — Additional file 3: Table 9. Calculated contraction ratio values (CR) between movement mode (concentric/eccentric) for all performed exercises and observed muscles (mean ± SD) in the four conditions (pipes). [file 13102_2020_168_MOESM3_ESM.docx]

**Appendix 2. (Supplementary material)**

| **Table 9.**  Calculated contraction ratio values (*CR*) between movement mode (concentric/eccentric) for all performed exercises and observed muscles (mean ± SD) in the four conditions (pipes). | | | | | | | |
| --- | --- | --- | --- | --- | --- | --- | --- |
| **Exercise** | **Condition** | **U.TA** | **DE** | **L.TA** | **LD** | **SA** | **PE** |
| **In/Ex rotation** | **P** | 0.61±0.48 | 1.05±1.09 | 0.26±0.20 | 1.83±1.14 | 0.39±0.32 | 5.89±5.67 |
|  | **PW** | 0.57±0.46 | 1.22±1.78 | 0.26±0.22 | 1.71±1.12 | 0.36±0.34 | 6.23±5.78 |
|  | **PG** | 0.59±0.51 | 1.15±1.28 | 0.23±0.16 | 1.88±1.26 | 0.34±0.28 | 6.03±4.66 |
|  | **PWG** | 0.61±0.48 | 1.18±1.37 | 0.24±0.17 | 1.80±1.22 | 0.36±0.37 | 6.80±6.47 |
| **Ab/Ad** | **P** | 0.32±0.37 | 0.39±0.29 | 0.19±0.10 | 3.39±3.26 | 0.40±0.31 | 6.14±4.79 |
|  | **PW** | 0.29±0.20 | 0.38±0.28 | 0.18±0.10 | 3.41±3.10 | 0.42±0.36 | 5.62±3.63 |
|  | **PG** | 0.26±0.18 | 0.31±0.23 | 0.16±0.10 | 3.07±3.45 | 0.35±0.28 | 5.99±4.22 |
|  | **PWG** | 0.32±0.36 | 0.36±0.23 | 0.17±0.09 | 3.56±3.57 | 0.40±0.28 | 6.23±5.29 |
| **diagonal F/E** | **P** | 0.32±0.30 | 0.44±0.35 | 0.21±0.15 | 3.46±3.75 | 0.46±0.37 | 5.46±4.74 |
|  | **PW** | 0.28±0.21 | 0.51±0.42 | 0.24±0.19 | 3.64±3.87 | 0.48±0.40 | 7.24±7.68 |
|  | **PG** | 0.34±0.26 | 0.46±0.41 | 0.23±0.19 | 3.84±4.49 | 0.44±0.42 | 7.14±8.60 |
|  | **PWG** | 0.38±0.29 | 0.49±0.36 | 0.27±0.18 | 4.03±3.88 | 0.43±0.26 | 5.77±4.71 |
| P: empty pipe (0.5 kg); PG: weight (stable mass; 4.5 kg); PW: water (unstable mass; 1 kg); PWG: water + weight (unstable mass; 4.5 kg); U/L .TA: upper/lower trapezius; DE: deltoid; LD: latissimus dorsi; SA: serratus anterior; PE: pectoralis major; Contraction ratio: concentric RMS (%MVIC)/eccentric RMS (%MVIC) | | | | | | | |
